# Extracellular Vesicles for Therapeutic Nucleic Acid Delivery: Loading Strategies and Challenges

**DOI:** 10.3390/ijms24087287

**Published:** 2023-04-14

**Authors:** Anastasiya Oshchepkova, Marina Zenkova, Valentin Vlassov

**Affiliations:** Institute of Chemical Biology and Fundamental Medicine SB RAS, 630090 Novosibirsk, Russia

**Keywords:** extracellular vesicles, drug delivery, nucleic acid therapeutics, nanocarrier, exosomes, apoptotic bodies, microvesicles, ectosomes, multivesicular body, intraluminal vesicles

## Abstract

Extracellular vesicles (EVs) are membrane vesicles released into the extracellular milieu by cells of various origins. They contain different biological cargoes, protecting them from degradation by environmental factors. There is an opinion that EVs have a number of advantages over synthetic carriers, creating new opportunities for drug delivery. In this review, we discuss the ability of EVs to function as carriers for therapeutic nucleic acids (tNAs), challenges associated with the use of such carriers in vivo, and various strategies for tNA loading into EVs.

## 1. Introduction

Therapeutic nucleic acids (tNAs)—DNA, RNA, and their chemically modified analogs [1,2,3,4,5,6,7,8,9]—represent a promising class of innovative therapeutics that require carriers for their delivery into cells/tissues [10,11,12,13,14,15,16,17]. Since the pioneer work published by M. L. Stephenson and P. C. Zamecnik in 1978 [18], tremendous progress has been achieved in the field of gene targeting, resulting in recent years in the development of the first antisense oligonucleotide (mipomersen, 2013, Kynamro), and the first small interfering RNA (patisiran, 2018, Onpattro) has been approved by the FDA (United States Food and Drug Administration) for clinical application. After that, several DNA- and RNA-based products have successfully passed through clinical trials and been approved for clinical use with a number of others being at different stages of preclinical and clinical investigations [19,20,21,22].

Similar to small molecule drugs and proteins, tNA-based therapeutics are sensitive to various environmental factors, among which nucleases are the most important, limiting tNA circulation time and bioperformance [23]. Nowadays, the problem of nucleic acid (NA) sensitivity to nucleases can be said to be solved using optimized modification patterns of the ribose/deoxyribose-phosphate backbone [24,25]. For example, the phosphorothioate backbone or 2′-carbon modifications are commonly used to improve tNA pharmacokinetics [2], affinity for targets, and protection from nucleases [5]. Another challenge limiting the wide application of tNAs is the inability of tNAs to cross the plasma membrane barrier of cells. This is why different carriers have been developed to improve the intracellular/intratissue accumulation of tNAs [26,27,28,29], including those proving for targeted delivery. Despite the considerable progress made in this field, major challenges remain to be overcome: some carriers cause immunological problems or are unable to overcome the biological barriers on their way to the target; some of them are rapidly excreted by the kidney or are accumulated intensively and metabolized by the liver.

Extracellular vesicles (EVs) and virus-like particles have raised great interest as delivery vesicles since they are naturally internalized by human cells. Viral carriers are highly effective for tNA transport, including drugs that need to be delivered into the nucleus [8]. However, they have serious limitations related to their immunogenicity and the risk of insertional mutagenesis. EVs have acceptable biocompatibility, although some challenges remain due to the lack of efficient technologies providing for their mass production. Remarkably, virus ligands are often used to engineer EVs. For instance, the vesicular stomatitis virus G protein (VSV-G) is applied to improve the endosomal escape of EVs [30]. According to recent calculations, only ~20% of EVs will be able to release their content into the cytoplasm if they are internalized by endocytosis [31]. 

The application of various EVs and EV mimetics for tNA delivery is detailed in comprehensive reviews [32,33,34,35,36,37,38]. Here, we focused on the challenges and the most promising approaches for loading tNAs into EVs and discussed some aspects of EV biogenesis that may be useful for designing the EV-based tNA delivery systems. We paid more attention to endogenous loading strategies as they distinguish EVs from other lipid-based carriers and can be their potential advantage. 

## 2. Classification of EVs

EVs are heterogeneous populations of membrane vesicles released by prokaryotic and eukaryotic cells into the extracellular milieu in response to stress, death, or during intercellular communication. These nanosized carriers transfer various cargoes into the body, protecting them from enzymatic degradation. The unique architecture and content of EVs have stimulated research aimed at their use as drug carriers, diagnostic markers, immunotherapeutic agents, and for regenerative medicine [39,40,41,42,43,44,45,46,47,48,49,50,51]. Two general pathways of EV formation have been described to date: via the intracellular endosomal compartments and via the separation of vesicles from the plasma membrane [52]. 

There are three main types of EVs: apoptotic bodies (~500–5000 nm and larger [53]), microvesicles, also called microparticles or ectosomes (~100–1000 nm), and exosomes (~30–150 nm) [52]. It should be noted that the International Society for Extracellular Vesicles [54] today proposes to use the terms small (<200 nm) and large EVs (>200 nm) instead of exosomes and microvesicles, respectively, since it is still impossible to distinguish individual types of EVs during analysis. In fact, there are no efficient methodological approaches that provide a reliable way to identify and study the individual types of EVs. This leads to problems with the interpretation of data. Here, we use the terms “exosomes” and “microvesicles”, implying that the population of small EVs is enriched by exosomes, while the population of large EVs is enriched by microvesicles in most cell types. 

The sizes and protein markers of the different types of EVs overlap [55]. Moreover, the protein composition of EVs varies depending on their cellular origin [56]. As it turned out, heterogeneity is one of the main characteristics of EVs because even the EV population secreted by an individual cell is heterogeneous [57]. Among numerous attempts to identify the criteria for separating EVs, an assay of tetraspanins plays an important role. It has been suggested that quantitative assessment of tetraspanins can help in the separation of exosomes and microvesicles of similar size, at least for some cell types [58,59]. In some cases, a significant enrichment of CD63 in the endosomes and CD9 in the plasma membrane was found [58,59]. Nevertheless, the problem of the physical separation of the different types of EVs remains to be solved. 

## 3. Challenges of EV Application as Drug Delivery Carriers

There are minimal requirements for carriers used for tNA delivery in the clinic. Briefly, they should provide for long circulation, low immunogenicity, and high intracellular accumulation of tNAs, simultaneously. In addition, the tNA delivery system must be biodegradable and act in a specific manner. A number of reviews have already discussed the use of EVs as a delivery system in various aspects: the EV surface molecules potentially involved in their interaction with recipient cells [60], the doses of EVs used for preclinical studies [61], strategies for engineering EVs with specific targeting ligands [62,63,64,65,66], approaches used for increasing the EV stability in the bloodstream [62,63]. For this reason, this section will focus on the issues that deserve special attention.

### 3.1. Interactions of EVs with Immune Cells

EVs are particles that are naturally secreted and taken up by cells. For this reason, these carriers are expected to be biocompatible. Indeed, EVs were shown to cause only negligible side effects on cell physiology or survival [67]. However, it is known that EVs make a significant contribution to the development of immune responses, especially if they originate from cells of the immune system. There are increasing pieces of evidence that EVs, released by nonimmune cells, can also participate in the development of immune responses [68]. Blood cells are often chosen for the production of EVs, which, as it has turned out, can stimulate inflammation [69,70]. EVs derived from other cellular sources may also cause a moderate proinflammatory response. Thus, SKOV3- or HEK293-derived EVs caused a slight activation of tumor necrosis factor-α (TNF-α) or interferon-α (INF-α) production by peripheral blood mononuclear cells (PBMCs) [71]. It seems that, regardless of the origin of EVs, they tend to have a visible effect on cells of the immune system, and such impacts have to be monitored in the case of long-term treatment.

The impact of nonimmune EVs on host immunity has been well illustrated by EVs isolated from cow milk and administrated to female Sprague–Dawley rats by oral gavage [72]. Today, a long-term treatment strategy with well-tolerated doses of therapeutics is preferred. Although this avoids acute reactions to therapy, the body develops chronic responses to exposure. After long-term medication (once daily for 15 days) with milk EVs (25 mg/kg b. wt), an increase in the level of granulocyte-macrophage colony-stimulating factor (GM-CSF) and a ~40% decrease in triglycerides were noted in the serum of healthy rats despite the absence of other serious changes [72].

Two models have been proposed to explain the action of milk EVs in the intestine. First, the uptake of EVs by enterocytes can cause the production of defective chylomicrons, which can be recognized and eliminated by intestinal macrophages. A more plausible explanation is that the macrophages engulf aggregates of chylomicrons and EVs. Recently, the associations of EVs and lipid particles, yielding aggregates, have been discussed [73,74,75]. In any case, the reduced level of triglycerides in the rat’s blood is easily explained by the macrophage-mediated elimination of chylomicrons from the intestine that prevents chylomicron movement into the lacteal (lymph capillary) and subsequent entry into the venous circulation [76].

Intrigue enough, these milk EVs (25 mg/kg b. wt.) had a long-term inhibitory effect (~4–5 weeks) on tumor growth in mice of human lung cancer (A549) xenografts exclusively after administration by oral gavage (three times a week), but not after intraperitoneal injection [72]. It looks like the action of EVs in the intestine was the origin of their therapeutic effect. The positive effect of EVs administered through the digestive tract was also confirmed by other observations [77].

### 3.2. EVs in the Bloodstream

Cancer is still one of the leading causes of death in the world, and EVs are considered promising carriers for therapeutics in oncology. Frequently, EVs are intravenously injected into the body based on the idea of their passive accumulation in a tumor site through the enhanced permeability and retention (EPR) effect [78,79]. However, the ability of EVs to overcome significant distances through the bloodstream seems questionable. Furthermore, the EPR effect seems to be overestimated in humans [78,79].

The first barrier on the way of EVs to the target sites (tumor) is blood phagocytes. A recent study showed that tumor EVs in the blood vessels of a zebrafish embryo were preferentially taken up by patrolling macrophages (functionally similar to human patrolling monocytes), endothelial cells, and putative hematopoietic stem cells [80]. An interaction of EVs with non-cellular blood components, such as low-density lipoproteins (LDLs) [73], also contributes to EV removal from the bloodstream. An electron microscopy assay of crude human plasma has shown that EVs frequently bind and fuse with lipoprotein-like structures [75]. In vitro data indicate that EVs secreted by highly malignant breast cancer cells metastasizing to the brain are able to form aggregates with LDLs, which can subsequently be taken up by monocytes [73].

Eventually, in addition to the removal of EVs directly from the blood, there is a risk of their accumulation in the reticuloendothelial-system-rich organs [31]. This could result in EVs failing to meet their targets. The use of high doses of EVs can lead to their intensive accumulation in the lungs that can cause asphyxia, as has been found in experiments with mice [81].

### 3.3. EVs as a Part of the Cellular Secretory Apparatus

**Figure 1:**
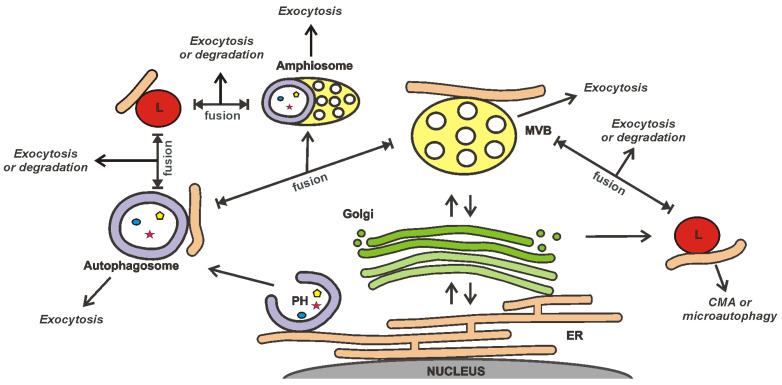
MVB interacts with other secretory and degradation processes in a cell. MVBs, autophagosomes, and amphisomes may undergo exocytosis or fusion with lysosomes, resulting in either degradation of their content or lysosomal exocytosis. It should be noted that some paths shown in the figure are hypothetical. The scheme was prepared on the basis of the reviews [82,84,88,89,90]. MVB—multivesicular body; PH—phagophore; CMA—chaperone-mediated autophagy; ER—endoplasmic reticulum; L—lysosome.

The release of EVs is closely integrated with other secretory and degradation processes in a cell (Figure 1) [82,83,84,85,86,87]. This integration is suggested by the results of studies demonstrating that the secretion of EVs can be increased to compensate for lysosomal impairment [84,86] or autophagy inhibition [83]. Sometimes, the loading of drugs into EVs can be performed using the cellular mechanisms of vesicle production. This technique is called “indirect”, “endogenous”, or “preloading”. A tNA or another cargo is added to cells, after which the secreted EVs are isolated. However, the intersections between EVs and other secretory/degradation processes inside cells can reduce the effectiveness of this drug loading strategy. Particular, the exocytosis of cargo can be expected to occur simultaneously via several secretory pathways; e.g., recently, the presence of overexpressed alpha-synuclein was simultaneously detected in endosomes, lysosomes, and autophagosomes [85]. 

The connection of EVs with other secretory/degradation processes may additionally provide new opportunities to control the EV release and the search for proteins suitable for selective loading of tNAs into EVs. An important question is the link between EV secretion and autophagy; e.g., the autophagy-associated protein Atg5 (autophagy protein 5) was found to control the release of EVs by the regulation of multivesicular body (MVB) acidification [91]. Atg5 decreases the activity of V_1_V_0_-ATPase by detaching its Atp6v1e1 component [91]. The deactivation of V_1_V_0_-ATPase reduces MVB acidity and promotes EV release. The sorting of Atp6v1e1 into intraluminal vesicles (ILVs) of MVBs depends on LC3 (microtubule-associated protein 1A/1B light chain 3). Most likely, the LC3 recruitment into ILVs is provided by Atg5. Eventually, both LC3 and Atp6v1e1 are released by cells via EVs [91].

From a practical point of view, LC3 can be of interest for the selective loading of tNAs into EVs. Its lipidated form, LC3-II, can be involved in ribonucleoprotein (RNP) sorting into ILVs [92]. LC3-II can recruit proteins containing the LC3-interaction region (LIR) [87], although it is not clear whether it is absolutely required for the sorting of RNA-binding proteins (RBPs) into ILVs [92]. Notably, LIR was not found in the previously mentioned protein, Atp6v1e1 [91]. The use of LC3-mediated loading of RNPs into ILVs can be of interest to those aiming at the therapeutic application of small nucleolar RNAs (snoRNAs) [92].

In addition to Atg5, there is growing evidence that proteins traditionally associated with autophagy can control MVB biogenesis. For instance, a recent report demonstrated the role of isoform A of lysosome-associated membrane protein 2 (LAMP2A) and heat shock 70-kDa protein 8 (HSC70) in the loading of proteins, containing the KFERQ-like motif, into EVs [93]. Although it is well known that both LAMP2A and HSC70 are involved in chaperone-mediated autophagy (CMA) [87,90], it is quite possible that, in combination with other factors, they can change the fate of cargoes, directing them to EVs. 

### 3.4. Influence of the Non-Cellular Environment on EVs

The interaction of exogenous EVs and cells is affected by various environmental factors. It has been found that the efficiency of EV internalization [94] and the biological effects mediated by EVs [95] can depend on the stiffness of the cell growth substrate; e.g., EVs secreted by highly malignant breast cancer cells can affect the activation, proliferation, motility, and contractility of fibroblasts only under conditions that mimic the stiffness of the tumor, but not healthy tissue [95]. 

A general recommendation for EVs designed for the delivery of therapeutics to tumor cells is to obtain EVs under conditions close to the tumor microenvironment. Thus, it was noted that EVs secreted by metastatic melanoma cells were more efficiently internalized by their own cells, when EVs were produced under the acidic pH of the growth medium (pH = 6.0) [96]. The rigidity of these “acidic” EVs was increased due to changes in the composition of the EV’s lipids [96]. Similarly, hypoxic tumor EVs were better internalized by cancer cells than normoxic EVs [97]. 

Sometimes, EVs show a greater affinity for their own cells [98,99]. In general, this is similar to how tumor cells prefer “acidic” or “hypoxic” EVs. In the case of tumor-derived EVs, this property of EVs has been proposed for use as a Trojan horse for drug delivery [99]. This trend may persist even after endogenous loading of cargo into EVs [100]. However, little is actually known about the effect of a cargo on the properties of EVs. Chemotherapeutic drugs can affect EVs after endogenous loading as they can influence the EV-secreting cells. It has been found that prolonged cell exposure to doxorubicin, sufficient for developing drug resistance, can change the protein profile of EVs secreted by these cells [101]. Usually, drug loading into EVs requires short-term treatment, which is expected to have no effect on the properties of EVs. 

In nature, the changes in the architecture of EVs can occur as a result of a viral infection. Viruses can penetrate inside EVs, thereby avoiding the host’s immune defense [102] and providing viral ligands on the surface of EVs [103]. Viruses need to control the EV composition to provide their entry into the type of cells that can ensure their propagation. Whether endogenously loaded cargo can affect the EV composition, such as viruses, is not entirely clear. The EV-mediated intercellular communication is believed to exist for targeted transfer of biological cargo from one cell to another [104]. It is reasonable to assume that cells should control the EV specificity depending on the type of cargo and the type of target cells.

### 3.5. Manufacture of EVs

The low yield of EVs is the key problem limiting their clinical applications. Although some external stimuli (e.g., cell starvation, hypoxia, or an increase in Ca^2+^ concentration in the culture medium) are used to increase the secretion of EVs [105], the composition and properties of such EVs can differ significantly from those obtained under normal conditions. This has recently been confirmed for EVs secreted by starving cells, where starvation significantly affects the protein composition of EVs [106]. The altering of the EV content in response to environmental stresses has also been discussed in a recently published review [107]. In addition to activating the EV secretion in stress conditions, it is possible to overexpress some proteins in EV-secreting cells, which can enhance the EV release into the extracellular space [108]. Although it may have a lesser influence on the content and features of EVs, the use of exogenous genetic constructs still raises concerns due to the risk of their inclusion into EVs, followed by insertional mutagenesis.

Isolation of EVs is considered a laborious and expensive process that is also poorly standardized. Conventional isolation approaches include ultracentrifugation, ultrafiltration, size exclusion chromatography, immunoaffinity, and polymer precipitation [109]. Among these, ultracentrifugation is the most commonly used technique [110]. Emerging technologies include membrane-based separation approaches and microfluidics [109]. Finally, large-scale production of EVs is based on the use of bioreactors [109,111]. A convenient classification of methods for EV isolation is based on the degree of purity and yield [112]. Summing up, the higher the degree of EV purification, the less the amount in the output. However, the low yield of EVs is not only a problem for highly purified EV preparations; even the standard ultracentrifugation protocol is not always sufficient to meet the quantitative requirements of laboratory studies.

Due to the different approaches used to isolate and purify EVs, there are significant inconsistencies in the doses of EVs used in preclinical studies [61]. The amount of EVs is usually measured either by the total protein concentration or by the particle number in the EV preparations (techniques of EV characterizations were discussed in reviews [40,50,113,114,115]). However, the different methods for isolating EVs give different levels of contamination in the EV preparations with nonvesicular structures. This may become the reason for the distortion in the quantification of EVs, followed by the diversity in the used therapeutic doses [61].

For clinical research, EVs are usually obtained from the recipients themselves, since the use of autologous EVs reduces the risk of EVs being recognized by the immune system. The EV recognition as potentially dangerous objects may lead to their accelerated clearance following administration. Recently, it was observed that repeated intravenous administration of Expi293F-derived EVs to non-human primates (*Macaca nemestrina*) may elicit the EV-specific antibody responses that result in the accelerated clearance of EVs [116]. Since the lifetime of primary cell cultures is restricted, it may not be possible to obtain enough amounts of EVs for a therapeutic dose. Thus, the main limitation of the clinical use of EVs is the absence of an optimal biological source for their production.

## 4. Strategies of tNA Loading into EVs

There are two ways for tNA loading into EVs. The first uses a cellular apparatus to sort a cargo into EVs and is called an indirect or endogenous loading strategy. The second is based on the loading of the previously isolated and purified EVs (direct or exogenous strategy). 

**Figure 2:**
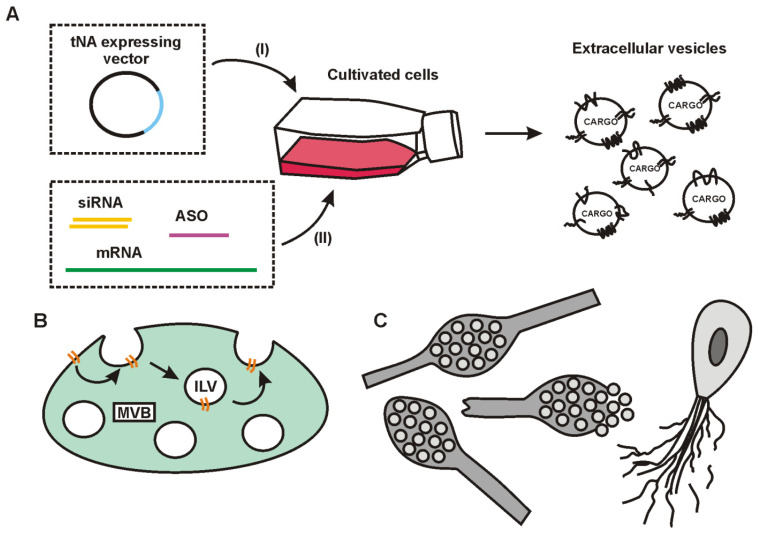
The endogenous strategies of tNA loading into EVs and schematic illustration of exosome and migrasome formation. (**A**) Endogenous strategies for tNA loading. The first (I) strategy is based on cell transduction, transfection, or electroporation by a tNA expressing vector. The second strategy (II) is based on the direct delivery of tNAs into EV-secreting cells. (**B**) Scheme of the ILV back-fusion. (**C**) The long tubular retraction fibers are formed by migrating cells during movement. Migrasomes localize on the tips or at the intersections of retraction fibers and contain numerous smaller (Ø 50–100 nm) vesicles. They are released into the extracellular environment after the fiber breaks. Apparently, in some cases, smaller vesicles inside them can exit into the extracellular space. ILV—intraluminal vesicle; MVB—multivesicular body; ASO—antisense oligonucleotide; siRNA—small interfering RNA.

From a practical point of view, the endogenous loading of tNAs into EVs seems very attractive. For packaging, tNAs are frequently overexpressed in EV-secreting cells (Figure 2A), but this procedure is shown to be of low efficiency [117]. Today, it is becoming clear that a high loading level of EVs with tNAs can be achieved through selective sorting, which means the use of cellular mechanisms providing specific enrichment of particular NA in EVs. In the current review, we pay attention to selective endogenous strategies, considering them in two main types of EVs of living eukaryotic cells: exosomes (Section 5) and microvesicles (Section 6). We also discuss apoptotic bodies, considering the possibility for their loading with tNAs (Section 7). Finally, in Section 8, we review endogenous strategies that can be used to load tNAs into EVs using their surface proteins, which are usually common to all types of EVs, or their belonging is not fully understood.

The direct (exogenous) strategies of tNA loading into EVs will be considered in Section 9. These approaches are based on manipulating the previously isolated and purified preparations of EVs and are based on physical or chemical exposures to EVs, and do not take into account the way of the EV formation inside cells.

Eventually, in Section 10, we discuss current clinical trials of EV-mediated delivery of tNAs, as well as some recent inventions in methods of tNA loading.

## 5. Exosome Loading with tNAs

To date, the main efforts in the endogenous loading strategies are focused on the application of exosomes for tNA delivery. This is because exosomes have a relatively small range in size compared with other types of EVs. Exosome secretion has been described for a number of eukaryotic organisms, but is still not well understood in detail. Today, it is becoming clear that the processes of exosome formation and the sorting of biological cargoes into them are closely interrelated. Understanding the mechanisms of exosome biogenesis and the sorting of NA-binding proteins into them is of decisive importance for developing the selective loading approaches. 

### 5.1. Basic Principles of Cargo Sorting into Exosomes and Exosome Release from Cells

Exosomes are EVs that are formed inside special cellular compartments called endosomes. Some endosomes may undergo “maturation”—a process accompanied by inward budding of the endosomal membrane. This leads to the formation of intraluminal vesicles (ILVs) inside them. Endosomes containing ILVs are referred to as multivesicular bodies (MVBs) (Figure 2B). Upon the fusion of MVBs with the plasma membrane, ILVs are released into the extracellular milieu [91,118,119]. Outside the cell, ILVs are called exosomes. 

It is important to note that the terms “ILVs” and “exosomes” are not used interchangeably because the fate of ILVs in a cell can be different. In addition to being secreted, ILVs can be destroyed upon MVB fusion with lysosomes [120,121], or MVBs can also be included in the autophagic secretion/degradation pathway (Figure 1). Finally, ILVs can follow the path of back-fusion (retrofusion) with the MVB membrane [122] (Figure 2B), although the existence of this mechanism is so far under debate. Recently, the release of large MVB-like structures has also been discussed [123]. A special mention is needed for migrasomes [124] that appear on the tips and at the intersections of retraction fibers of migrating cells [125] (Figure 2C). Although migrosomes look like MVBs, the nature of the enclosed vesicles in them remains unknown. Meanwhile, migrasomes seem to be involved in cell-to-cell communications since when one cell releases them, they can be taken up by another [125]. The contribution of migrasomes or vesicles enclosed in them in the total pool of secreted EVs is currently unknown. 

The biogenesis of exosomes inside cells begins with the generation of ILVs in endosomes. This process can be divided into three steps: (i) cargo binding with the endosomal surface (at least for proteins), (ii) bending of the endosomal membrane and formation of a bud of ILV, and (iii) cleavage to yield ILVs in the endosomal lumen. The stages of exosome formation inside cells are regulated by the endosomal sorting complexes required for transport (ESCRTs) and lipids [126,127,128,129,130,131,132,133,134,135,136,137,138,139,140,141,142]. Here, the human ESCRT proteins will be indicated by default in the text (according to [134]). ESCRTs are responsible for the formation of at least some populations of exosomes. ESCRT-independent mechanisms of exosome formation will not be discussed here.

The ESCRTs were divided into three complexes, designated by roman numerals from I to III [134]. Additionally, the complex that is required to recruit ESCRT-I to the endosomal membrane is referred to as ESCRT-0 [134]. The assembly and dissociation of the ESCRTs on/from the endosomes occur by concerted waves [143,144]. Disagreements still exist on the definition of the complexes responsible for generating the endosomal membrane deformation. Most likely, the bending is governed either by ESCRT-I and -II [145,146] or by ESCRT-III and ATPase VPS4 [147,148]. One of the hypotheses suggests that the upstream ESCRTs (0-II), together with sorted cargo, crowd in a microdomain of the endosomal membrane, leading to its initial deformation [146]. Another model proposes that VPS4 ATPase draws together ESCRT-III filaments on the endosomal membrane, thereby mediating the crowding of endosomal cargo and subsequent inward membrane bending [147]. Notably, both mechanisms are consistent with the fact that endosomal membrane invagination is primarily mediated by the concentration of endosomal cargo. Finally, there seems to be no doubt that the scission of the bud of ILVs is governed by ESCRT-III and VPS4.

The process of exosome release from cells is facilitated by the fusion of MVBs with the plasma membrane. Recent observations indicate that less acidic MVBs will be exocytosed [119]. It turned out that neutral sphingomyelinase 2 (nSMase2), involved in ceramide biogenesis, may control exosome secretion by counteracting V-ATPase-mediated acidification of MVBs (also known as vacuolar or V_1_V_0_-ATPase) [119]. The role of SNARE [127,129,130,132,136], Rab [149,150,151], and Ral [152] proteins in MVB transport and fusion with the plasma membrane was also established. Rabs govern the intracellular motility of endosomes via the regulation of microtubule-associated proteins for long-range movement or actin-dependent proteins for short distances [153]. Recently, an actin-binding protein, cortactin, its antagonist, coronin1b, fascin-1, and Rab27a were identified as participants in the MVB docking process [154,155]. However, it is noted that fascin-1 can act independently of its actin-binding function [155]. Today, the placement of the MVB docking sites in invadopodia is widely discussed [154,155].

Apparently, there are different independent pathways of exosome biogenesis. This is especially noticeable in cells with clearly defined apical–basolateral asymmetry. For instance, in MDCK canine kidney cells, the basolateral secretion of exosomes may depend on ceramides, whereas on the apical surface, the exosomal release is mediated by ALIX (ALG-2-interacting protein X) [156]. In human cholangiocytes, ceramides and ALIX appear to regulate the release of exosomes from both the apical and basolateral surfaces of cells [157]. ALIX is also likely to control the apical secretion of microvesicles [157]. It has been proposed to consider different MVBs as a source of exosome heterogeneity in polarized cells [156]. By the way, Rabs involved in the regulation of the basolateral and apical secretions of exosomes in MDCKs have recently been identified [151].

### 5.2. Involvement of RNA-Binding Proteins in tNA Sorting into Exosomes

Unfortunately, we are forced to ignore the issue of the selective sorting of cellular DNA into exosomes, since this process is poorly understood and remains debatable. Endogenous sorting of RNA into exosomes occurs during their formation within endosomes and appears to be mediated by two main events: (i) RNA binding with RBPs [158,159] and (ii) RNA affinity to endosomal lipids [160,161].

RBPs bind RNA and transport it to the endosomal membrane. The accumulation of RNPs on the surface of endosomes stimulates the formation of ILVs. In addition, RNA potentially has its own affinity for endosomal lipids [160,161] that can also facilitate its sorting into ILVs. Upon the fusion of MVBs with the plasma membrane, exosomes containing RNPs are released into the extracellular space. 

One of the well-studied RBPs is Y-box binding protein 1 (YB-1 or YBX1) [162]. YB-1 regulates the sorting of various RNAs into exosomes [163,164,165,166], and similar to many other RBPs, YB-1 has an affinity for specific nucleotide sequences [165,166]. The search for RNA motifs recognized by different RBPs has received a lot of attention currently due to the possibility of manipulating or stimulating the active loading of tNA cargo into exosomes. However, it is worth noting that the sorting of some RNA by RBPs may be associated with the recognition of the secondary structure of RNA rather than specific nucleotide motifs [164]. 

It was shown that YB-1 can form liquid-like condensates in cells due to the presence of an intrinsically disordered region in its structure [163]. Cellular membraneless organelles are often composed of both RNA and proteins [167]. Notably, many proteins that undergo LC3-dependent sorting into exosomes are found in such structures, namely, in the stress granules and the P (processing) bodies [92]. Complexes of miRNA and YB-1 most likely also enter the endosome from the P-bodies [163], where the endoplasmic reticulum can be an intermediator between them [168].

### 5.3. Upstream ESCRTs (0-II)-Mediated Binding of RBPs and RNA

Our knowledge of endosomal cargo sorting is based initially on the study of the biogenesis of transmembrane receptors, which can be removed from the cell surface through endocytosis. The conventional model describes the participation of the upstream ESCRTs (0-II) in the sorting of ubiquitinylated proteins into endosomes. Hepatocyte growth factor-regulated tyrosine kinase substrate (HRS) and signal transducing adaptor molecule 1/2 (STAM1/2) are subunits of ESCRT-0. They act synergistically [169] and bind ubiquitin-modified proteins through ubiquitin-interacting sites. ESCRT-0 accumulates on early endosomes via the phosphatidylinositol 3-phosphate (PI3P)-binding FYVE (Fab 1/YOTB/Vac 1/EEA1) domain of HRS [170]. Coiled-coil domain 2 (CC2) also directs HRS to early endosomes [170] and mediates its interaction with STAM1/2 [171]. 

The participation of HRS and STAM1/2 in the sorting of proteins into exosomes is usually not considered. However, a recent study have shown that, under inflammation, HRS can bind to RBP fragile X messenger ribonucleoprotein 1 (FMR1, also named fragile X mental retardation protein 1), which selectively loads miRNAs into exosomes by interacting with the short 5-nt sequence AAUGC [172]. 

Among other upstream ESCRTs, ESCRT-II has been identified as an mRNA-binding protein complex [173]. However, its RNA-binding function does not appear to be related to its activity in endosomes [174]. Thus, clarification of the role of ESCRT-0-II in the sorting of RNA and RBPs into exosomes is just beginning to emerge.

### 5.4. ALIX, Syntenin-1, and Syndecans Participate in tNA Sorting into Exosomes

**Figure 3:**
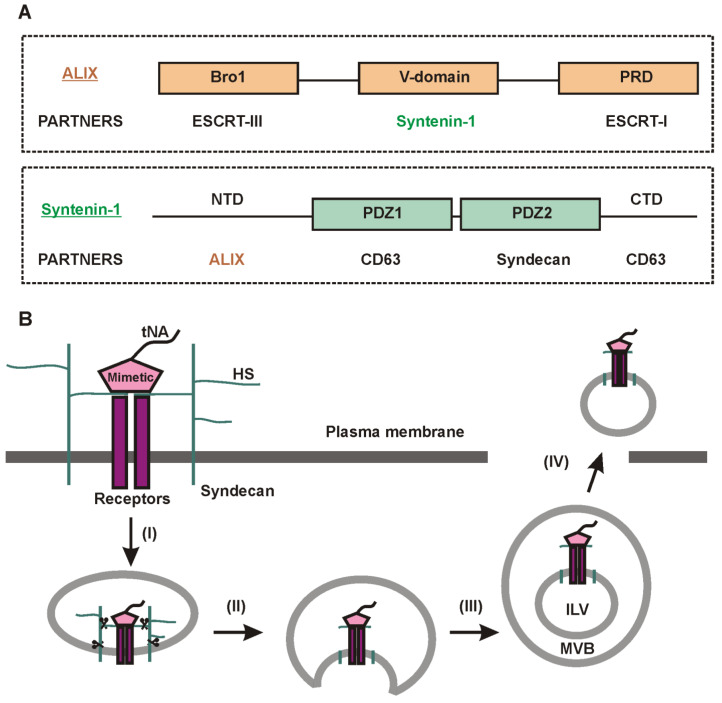
The ALIX–syntenin–syndecan pathway of exosome biogenesis. (**A**) Schematic illustration of the organization of ALIX and syntenin-1 protein domain structures [175,179]. (**B**) A proposed scheme for loading a tNA-conjugated ligand mimetic into exosomes. After endocytosis, syndecan is trimmered by heparanase in endosomes and subjected to proteolytic cleavage to its C-terminal fragment (I) [142,178,184]. The mimetic/receptor/HS complex can be sorted into ILVs in an ALIX- and syntenin-1- dependent manner (II–III; ALIX, syntenin-1, and ESCRTs not shown). The complex can be secreted into the extracellular environment via exosomes (IV). HS—heparan sulfate; PRD—proline-rich domain; NTD—N-terminal domain; CTD—C-terminal domain.

ALIX (ALG-2-interacting protein X) is a multifunctional protein containing the Bro1 domain (Figure 3A) [175,176]. ALIX was found to be one of the most reliable protein markers of exosomes [177]. There is an opinion that ALIX, together with syntenin-1 and syndecan, is involved in the formation of a separate population of exosomes [142,178]. ALIX binds to the LYPX(n)L motifs present in the N-terminal domain of syntenin-1 [142,178]. In turn, syntenin-1 interacts with multiple proteins through its two PDZ (PSD95/Dlg/ZO-1) domains [179] (Figure 3A). Its partners include syndecans [142,178], as well as tetraspanin CD63 [180,181]. Moreover, syntenin-1 has been shown to interact directly with phosphatidic acid in the endosomal membrane [182], making it a potential regulator of exosome biogenesis [141]. 

Some tNAs cannot be loaded into exosomes through expression in cells (Figure 2A, I). In particular, chemically modified tNAs must be delivered to EV-secreting cells in a ready-to-use form (Figure 2A, II). In addition to the use of intracellular mechanisms for sorting tNAs into exosomes, syndecans, which are located on the cell surface, may be of interest for tNA sorting and loading. Syndecans contain negatively charged heparan sulfate (HS) chains [183,184]. Therefore, their direct interaction with tNAs is restricted by electrostatic repulsion. However, it is known that HSs form ternary complexes on the cell surface with various ligands and their receptors, which can be potentially subjected to endocytosis and then be included in exosomes [178]. In theory, these ligands in the ternary complexes can be replaced by mimetics conjugated with tNAs (Figure 3B). 

Several growth factors that can interact with HS have recently been developed based on peptides or aptamers. Thus, the single-chain tandem macrocyclic peptides (STaMPtides) were designed to replace hepatocyte growth factor (HGF) [185]. STaMPtides are convenient to use because they can be obtained by the expression in bacterial cells. In addition, attempts were made to create DNA aptamers to mimic the basic fibroblast growth factor (bFGF) [186]. The actions of both the STaMPtides and the DNA aptamers were aimed at the stimulation of receptor dimerization. Following activation, receptors are typically subjected to endocytosis. 

We are not aware of any reports in which growth factor mimetics have been used to mediate tNA sorting into exosomes. Therefore, it is not known how the conjugation of mimetics with tNAs may affect their functionality. Moreover, it is not clear whether the ligand/receptor/HS complex is retained in exosomes in its original form. Finally, such strategy is able to bind a therapeutic cargo only with the surface of exosomes.

### 5.5. Some Aspects of mRNA Loading into Exosomes

Endogenous loading of mRNA into exosomes can occur through the strategies mentioned above (Figure 2A): using the tNA-expressing vector or a transcript prepared in vitro. Although the endogenous expression has many advantages, there is a possibility that plasmid DNA (pDNA) will be packaged into exosomes along with mRNA. In general, this is considered undesirable because the delivery of pDNA is associated with the risk of insertional mutagenesis.

Transcripts of mRNA can be prepared in vitro by enzymatic or chemical synthesis. These two approaches are often compared with regard to the inclusion of modified nucleotides into mRNA. Modifications can increase the mRNA nuclease resistance and reduce the risks of activation of the pattern recognition receptors [187]. The disadvantage of enzymatic synthesis is the impossibility of the selective inclusion of modified nucleotides into the mRNA sequence. Notably, the changes in the structure of mRNA can cause the loss of its functionality. Chemical synthesis, while allowing the production of the mRNA of any architecture, cannot provide for the production of long nucleotide sequences.

Overall, only a few approaches have been developed for the selective loading of mRNA into exosomes: one of them is used as a special nucleotide sequence “zipcode” to stimulate the mRNA loading [188]. This is a stem-loop-forming sequence of 25-nt (5′-ACCCTGCCGCCTGGACTCCGCCTGT-3′), variations of which are present in the 3′UTR of some mRNA enriched in exosomes. The zipcode contains a short “CTGCC” core sequence and a miRNA-1289 binding site. It is proposed that the interaction of miRNA-1289 with zipcode regulates mRNA transport into exosomes [188].

The zipcode was used to package *HchrR6* mRNA into exosomes. HChrR6 is an improved bacterial enzyme that converts CNOB (C_16_H_7_CIN_2_O_4_) into the cytotoxic drug MCHB (C_16_H_9_CIN_2_O_2_). To load *HchrR6* mRNA into exosomes, two strategies were used. First, the mRNA-expressing plasmid was developed: two tandem copies of the zipcode sequence were inserted at the 3′UTR of the *HchrR6* gene; the plasmid was used for the transient transfection of EV-secreting cells, followed by collecting the *HchrR6* mRNA-bearing exosomes [189]. Another approach was based on the delivery of in vitro transcribed *HchrR6* mRNA into EV-secreting cells using polyethyleneimine (PEI). Surprisingly, no difference was found in the loading efficiency of *HchrR6* mRNA prepared in vitro with or without the zipcode sequence [190]. In general, however, the endogenous packaging of in vitro transcribed *HchrR6* mRNA turned out to be more profitable than the packaging of the same zipcode-containing mRNA expressed intracellularly. In the former case (delivery into cells of the in vitro prepared *HchrR6* mRNA transcript), ~100 times less exosomes were required to donate one mRNA copy [190].

## 6. Microvesicle Loading with tNAs

**Figure 4:**
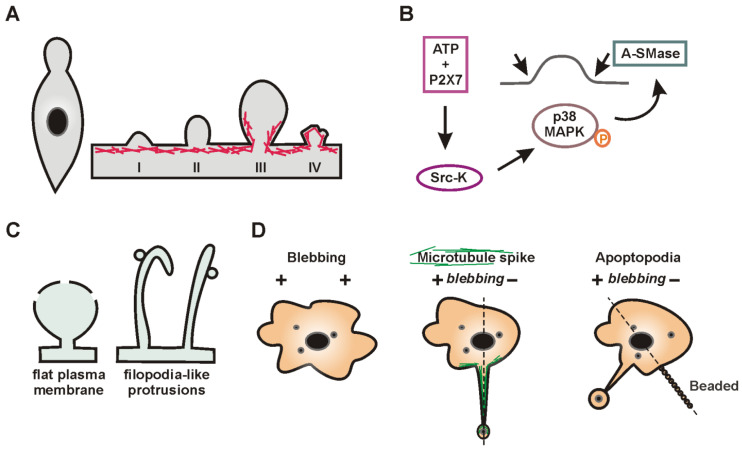
The formation of blebs and similar structures on the plasma membrane. (**A**) A characteristic feature of the amoeboid movement is the formation of a bleb lacking the actin cortex (I) [191]. Its size expands according to the influx of the cytoplasm (II). The bleb growth stops when the actin cytoskeleton (red lines, III) reassembles. Eventually, the bleb retracts into the plasma membrane (IV). (**B**) Receptor-induced biogenesis of microvesicles. Stimulation of the P2X7 receptor by ATP results in the phosphorylation of P38 MAP kinase (p38 MAPK) by src-protein tyrosine kinase (Src-K). Phosphorylation of P38 promotes the translocation of acid sphingomyelinase (A-SMase) to the outer leaflet of the plasma membrane, where it triggers ceramide-dependent microvesicle biogenesis [197]. (**C**) Vesicle shedding is a form of the plasma membrane repair mechanism [193]. Annexins have a high affinity for PS and play a key role in the removal of injured membrane regions. Annexin A7 (ANXA7) interacts with the ALG-2 (apoptosis linked gene-2) protein, which is a partner of ALIX. In turn, the ESCRT-III complex is assembled through ALIX and provides the damage site shedding [134,193]. In some cases, the vesicularization is preceded by the formation of filopodia-like protrusions at the damage site [195]. (**D**) Formation of apoptotic bodies [205]. Microtubule-rich membrane protrusions (microtubule spikes) and beaded apoptopodia can be formed in the presence or absence of membrane blebbing. Thin, string-like apoptopodia are generated between membrane blebs.

Microvesicles are a population of EVs that form in a cell by the outward budding of the plasma membrane. These EVs are still rarely used for tNA delivery due to a wide range of their sizes compared with exosomes. The diversity of processes that may contribute to the formation of microvesicles or microvesicle-like structures suggests that they may be even more heterogeneous than exosomes. The formation of the plasma membrane blebs or similar structures occurs during various biological processes (Figure 4): cell movement [191,192], repair of the plasma membrane [193,194,195], or stimulation of microvesicle release after ligand-mediated activation of some transmembrane receptors [196,197]. The fate of these protrusions may be different. For example, during amoeboid migration, blebs are used by cells as supports, followed by their subsequent retraction. Conversely, microvesicles and apoptotic bodies are shed from the cell surface into the environment. Microvesicles may be shed from the “flat” plasma membrane or from various protrusions [198]. 

The localization of phosphatidylserine (PS) in the outer leaflet of the microvesicle membrane is their characteristic feature [199], which can play an important role in maintaining their biological properties [200,201], and may be useful for tNA binding with their surface [202]. The placement of PS and phosphatidylethanolamine (PE) in the outer leaflet of the plasma membrane occurs during microvesicle biogenesis inside cells [203,204]. Usually, microvesicle formation involves a stage with an increased concentration of intracellular Ca^2+^, the source of which (intracellular or extracellular) has not been fully defined [203]. Calcium triggers a series of events that disrupt the connection of the plasma membrane with the cytoskeleton at the site of vesicle shedding. For example, calcium-activated calpain moves to the plasma membrane, where it participates in the remodeling of the cytoskeletal proteins; calcium also modulates the activity of membrane lipid translocases, promoting the placement of PS and PE in the outer leaflet of the plasma membrane [203,204]. 

Recently, a PS-mediated method for tNA binding with the surface of microvesicles has been proposed. The PS-binding motif of glyceraldehyde-3-phosphate dehydrogenase (GAPDH) was fused to the double-stranded RNA-binding domain of TARBP2 (TAR RNA-binding protein 2) and used to bind siRNA with PS, located on the outer leaflet of the microvesicle membrane [202]. This chimeric protein was expressed in bacterial cells, purified, and coincubated with EVs. Subsequently, it was improved to overcome endosomal entrapment by the attachment of endosomolytic peptides. Despite the fact that this approach implies the siRNA binding on the surface of microvesicles, it is assumed that TARBP2 can protect siRNA from degradation. The systemic administration of siRNA targeted to huntingtin gene (*Htt*) mRNA bound with brain-targeted microvesicles via the GAPDH–TARBP2 approach provided an almost 40% decrease in *Htt* mRNA, at least in the brain cortex of mice, modeling Huntington’s disease. The targeting of microvesicles to the brain was achieved by their modification with the RVG (rabies virus glycoprotein) peptide. 

In addition to PS, proteins involved in the intracellular sorting of cargoes into microvesicles can be used to develop new approaches for selective tNA loading. Microvesicle biogenesis is regulated by at least three GTPases: Cdc42 (cell division control protein 42 homolog) [206], RhoA (Ras homolog family member A) [207], and ARF6 (ADP-ribosylation factor 6) [208,209]. ARF6 is involved in the sorting of various cargoes into microvesicles [210]: proteins [211] and pre-miRNAs [212]. In addition, caveolin-1 has recently been identified as a regulator of the miRNA sorting into microvesicles; its action is due to interaction with RBP [213,214,215]. However, we are not aware of any reports where ARF6 or caveolin-1 was used to load tNAs into microvesicles.

A separate subtype of microvesicles, containing ARRDC1 (arrestin domain-containing protein 1), is distinguished [210] and used for tNA carrying [216]. These vesicles are sometimes referred to as ARMMs (ARRDC1-mediated microvesicles). The details of their biogenesis are not fully understood; however, the association of the ARRDC1 protein with ESCRTs is well known [217]. Apparently, the PSAP motif, located at the C-terminus of ARRDC1, binds TSG101 (tumor susceptibility gene 101 protein) (ESCRT-I) and transfers it to the plasma membrane [218,219]. Subsequently, the abscission of ARRDC1-containing microvesicles appears to be regulated by the VPS4 ATPase [219]. In addition to ARRDC1, the ARRDC4 protein may participate in the regulation of microvesicle biogenesis [220,221]. However, its mechanism of action is most likely not related to ESCRTs [220]. 

ARRDC1 can provide the endogenous packaging of therapeutic cargoes into microvesicles [216]. Thus, mRNA loading was performed by the fusion of a short Tat (transactivator of transcription) peptide to the C-terminus of ARRDC1, and the TAR (transactivating response) RNA element was fused to the 5′ end of mRNA [216]. TAR and Tat can bind specifically, providing mRNA loading into microvesicles. Through the binding of Cas9, ARRDC1 may provide the packaging of Cas9/sgRNA RNPs. For that, Cas9 was fused with the WW domains that have natural affinity for the PPXY motifs of ARRDC1 [216].

Apart from the PS- and ARRDC1-mediated approaches, we are not aware of other selective endogenous strategies for binding/loading of tNAs into microvesicles. Meanwhile, the use of microvesicles can be of great interest for the delivery of large nucleotide sequences. It has been shown that microvesicles have a clear advantage over exosomes in terms of pDNA delivery [222]. Meanwhile, the size of tNAs makes a significant contribution to the efficiency of microvesicle loading. For example, the loading of minicircle DNA into microvesicles was about twice as efficient as the loading of pDNA, which was two times larger than minicircle DNA [223]. 

## 7. Apoptotic Body Loading with tNAs

Apoptosis is a form of programmed cell death. Initiated by external or internal signals, it is regulated by a group of cysteine-aspartic proteases called caspases [224,225,226,227]. Apoptosis does not stimulate inflammation due to the preservation of the integrity of the cell membrane and the rapid elimination of cell fragments by macrophages or other phagocytes [224,225,226,227]. Sometimes, apoptotic cells are eliminated in vivo before being disassembled into apoptotic bodies [224,225]. In most cell cultures, apoptotic cells lose their integrity over time (secondary necrosis) due to the inability to remove dead cells or their fragments from the medium [224,226].

The disassembly of apoptotic cells into apoptotic bodies is preceded by a plasma membrane blebbing [225,228]. Interestingly, the formation of more complex cytological structures, such as microtubule spikes or apoptopodia, can be observed in some cells (Figure 4D) [205,225,228]. Such protrusions can be involved in the interaction of apoptotic cells and phagocytes [205,229] or in the generation of apoptotic bodies together with or independently of membrane blebbing [205]. The blebbing of apoptotic cells is regulated by the action of several kinases, including ROCK1 (Rho-associated kinase 1), LIMK1 (Lim domain kinase 1), and PAK2 (p21-activated kinase 2), activated by caspase-3 [205,228,230]. However, the key role of ROCK1 has recently been highlighted [230]. Caspases also regulate PS externalization in apoptotic cells by activating Xkr8 (Xk-related protein 8) scramblase [231], which forms heterodimers with Basigin (BSG) or Neuroplastin (NPTN) proteins in the plasma membrane [232,233]. In turn, PS-bound chemokines can attract phagocytes [234] to eliminate apoptotic bodies.

Apoptotic bodies are increasingly considered to be functionally important participants in intercellular communication [205,225,228,235]. It was previously assumed that their content could be formed stochastically. However, now it is believed that this may not always be the case. Perhaps, at least, the distribution of DNA and mitochondria in apoptotic bodies may depend on the mechanisms of cell disassembly [236]. In addition, the PANX1 (pannexin 1) protein was recently identified as a negative regulator of nuclear content distribution in apoptotic bodies [237]. All this indicates complex organization and regulation of the internal composition of apoptotic bodies. 

The variability in the size of apoptotic bodies, as well as their short lifetime (at 37 °C) [53,238], raises doubts about their real prospects for the transport of therapeutics. There is also concern about the safety of their use due to their origin from dying cells. On the other hand, heterogeneous populations of EVs released during apoptosis [239] may have different stabilities. It has recently been proposed to consider EVs, secreted by apoptotic cells, less than 1 µm in diameter as apoptotic microvesicles and from 1 to 5 µm in size as apoptotic bodies [235]. However, later, the size limits for apoptotic bodies were expanded [53]. The release of exosome-like vesicles by apoptotic cells has also been mentioned. Autolysosomes have been identified as the site of their formation in serum-starved endothelial cells [240]. It turned out that at least some exosome-like vesicles can be formed as an invagination of the inner side of the autolysosomal membrane, while their exocytosis is regulated by caspase-3 [240]. It is noteworthy that since various forms of cell death are often interrelated with each other, the classification of EVs, secreted by apoptotic cells, seems to be a difficult task. Moreover, it is possible that the release of EVs is a common feature of dying cells and is not exclusive to apoptosis [241]. 

In practice, obtaining large quantities of EVs can be achieved under conditions that are different from normal. Resting healthy eukaryotic cells secrete EVs at a low level; increased secretion occurs as a cell response to stress, illness, or death [206,242]. Apoptotic cells secrete significantly more EVs than living ones [243,244]. Apoptotic cells include all their content into apoptotic bodies, which is advantageous in the case of using the endogenous strategy of tNA loading. In this way, it was recently proposed to load phosphorothioate ASOs into apoptotic bodies designed for the delivery of their cargo to the brain [245]. The manufacture of ASO-containing apoptotic bodies was carried out in two steps: ASOs were combined with cationic konjac glucomannan (cKGM) and transferred into EV-secreting cells; cells were subjected to apoptosis by UV radiation and treatment with H_2_O_2_. Apoptotic bodies containing cKGM/ASOs (CABs) were generated by three types of mouse cells: 4T1 (mouse breast cancer cells), B16F10 (mouse melanoma cells), and ANA-1 (immortalized mouse peritoneal macrophages). It turns out that melanoma-derived CABs were the most promising for brain-targeted delivery [245]. Taking into account the degradation processes accompanying cell disassembly during apoptosis [228], only nuclease-resistant tNAs should be included in apoptotic bodies. 

It should be noted that apoptotic bodies are already used as delivery systems [246,247,248,249], but with some limitations. First of all, they are efficiently taken up by phagocytes due to the presence of PS on the outer leaflet of their membrane. Thus, they require “masking” or local administration. On the other hand, if phagocytes are the therapeutic targets, apoptotic bodies can be successfully used. Apoptotic bodies may create encouraging prospects for regenerative medicine due to their ability to trigger macrophage polarization towards the M2 phenotype [250]. Thus, in the mouse skin wound healing model, the therapeutic effect of locally used exogenous bone-marrow-derived mesenchymal stem cells embedded in hydrogel was provided by their apoptosis and the secretion of apoptotic bodies [250]. Apoptotic bodies were probably engulfed by F4/80-positive macrophages, promoting macrophage polarization towards the M2 phenotype. While this property may be useful for tissue repair, it may impose some limitations on the applicability of apoptotic bodies as drug delivery carriers. For example, the appearance of M2 macrophages in the immunosuppressive environment of a tumor may be undesirable. 

Another promising class of carriers is ghosts prepared from apoptotic bodies [251,252]. Ghosts are apoptotic bodies with removed internal content. They are often used to cover another carrier bearing a therapeutic drug. For example, ghosts from activated apoptotic T cells have been used to coat mesoporous silica nanoparticles (MSNs) preloaded with miRNA-21 mimic or curcumin. These chimeric apoptotic bodies (cABs) were used to modulate inflammation via the promotion of M2 polarization in macrophages [251]. It has been suggested that the membrane of apoptotic bodies, prepared from the activated T cells, confers the ability of cABs to target the inflammatory region. Because the membrane of the ghosts still contains the PS on the outer leaflet, the cABs are efficiently engulfed by macrophages. 

Since cABs are prepared based on apoptotic bodies, they also cause the aforementioned concerns related to their undesirable immunosuppressive features. However, it turned out that their potential to polarize macrophages towards the M2 phenotype was mainly observed in vitro. In a mouse cutaneous inflammatory wound model, cABs themselves promoted regeneration, but the rate of wound closure was significantly lower compared with the effect of cAB-mediated miRNA-21 or curcumin delivery. Moreover, the increased level of M2 macrophages (CD206 positive) in the skin was observed only in groups treated with cAB-miRNA-21 or cAB-curcumin [251]. In the dextran sulfate sodium (DSS)–induced colitis model, cABs themselves had a low, if any, positive therapeutic effect. They slightly reduced the concentrations of TNF-α in the mice’s serum, and practically did not change the levels of interleukin-6 (IL-6), transforming growth factor-β (TGF-β), and IL-10 [251]. Thus, the cAB-mediated immunosuppressive effects seem to be weak in vivo. Taken together, cABs look promising for delivering tNAs into phagocytes.

Our experience in studying biomimetic vesicles that mimic apoptotic bodies showed that they can be successfully used to deliver short oligonucleotides to tumor blood cells [253], at least under in vitro conditions. We observed that the delivery of DNA oligonucleotides by artificial cytochalasin B–induced membrane vesicles (also named cytochalasin B–inducible nanovesicles) to K562 cells was inefficient when they were generated by live cells. On the contrary, the use of cytochalasin B–induced membrane vesicles released by cells, pretreated with H_2_O_2_, provided almost 10-fold enhanced internalization of the oligonucleotide cargo by K562 cells. It is worth mentioning that blood cells represent one of the most difficult objects for tNA delivery.

## 8. The Use of Surface Proteins of EVs for Endogenous Loading of tNAs

Today, the most frequently used approaches for endogenous loading of tNAs into EVs are based on the use of EV surface proteins, since their engineering is understandable and technically easy to implement. However, it should be taken into account that the belonging of these proteins to a certain type of EVs has usually not been confirmed, or the protein is common to all of them.

Tetraspanins are most commonly used to load tNAs into EVs. These are transmembrane proteins that regulate different cellular processes via binding with various transmembrane and intracellular proteins. Tetraspanins have four transmembrane domains, an extensive extracellular region, forming two loops, a small inner loop, and C- and N-termini located in the cytoplasm [254]. The cytoplasmic tails of tetraspanins are usually modified to ensure selective sorting of cargo into EVs. 

Tetraspanins have become widely used as tools for editing the content of EVs since the vesicular localization of some of them is not in doubt (e.g., CD63, CD9, or CD81). It is important to emphasize that not all tetraspanins are positive markers of EVs. In particular, tetraspanin-6 acts as a negative regulator of EV secretion [120].

Tetraspanins are often used to load the RNA-guided CRISPR/Cas9 genome editing system into EVs [255]. CRISPR/Cas9 components can be packaged into EVs as an RNP, consisting of the Cas9 protein and a single-guide RNA (sgRNA). The delivery of CRISPR/Cas9 components in the form of RNP is considered to be safer than the use of pDNA or mRNA. The Cas9 protein and sgRNA have a natural affinity for each other. Therefore, it is enough to bind one of them to tetraspanin to load them into EVs. 

A specific interaction between an aptamer sequence attached to sgRNA and an aptamer-binding protein (ABP) fused with CD63 provided selective enrichment of EVs with Cas9/sgRNA RNPs [30]. A functional assay of gene editing activity indicated that the presence of ABP at both termini of CD63 was preferable. Another strategy was to load Cas9/sgRNA RNPs into EVs by constructing fusion proteins for CD63 with the green fluorescent protein (GFP) and Cas9 protein with GFP-binding nanobody (antibody) [256]. The system containing GFP-binding nanobody provided significantly higher levels of sgRNA, Cas9 protein, and Cas9-encoding mRNA in EVs and, as a consequence, increased the editing activity of EVs with respect to nonfunctional DsRed fluorescent protein, whose expression in A549 cells was blocked by the presence of a stop sequence. Recently, inducible loading systems with the participation of tetraspanin CD9 have been studied [257]. CD9 ensures the recruitment of a high level of Cas9 into EVs. However, neither Cas9 nor Cas9/sgRNA loaded into EVs via CD9 was functional.

In addition, tetraspanins can ensure the incorporation of various types of RNA into EVs by their fusion with RBPs. Thus, the human antigen R (HuR) fused with CD9 can mediate the sorting of RNA containing AU-rich sequences into EVs [258]. CD9-HuR greatly enriched AU-rich miRNA-155 in EVs. In contrast, it had no effect on the enrichment of AU-poor miRNA-328. CD9-HuR provides the loading of dCas9-encoding mRNA into EVs due to the insertion of three AU-rich sequences downstream of the stop codon. Another research was based on the creation of a fusion protein for CD9 and the RNA-binding protein AGO2 (hCD9.hAGO2) [259]. Similar to the use of CD9 in the inducible loading systems [257], CD9 provided the enrichment of some small RNAs (miRNA or shRNA) in EVs; however, there was no biological effect after the EV-mediated delivery of tNAs into recipient cells. Therefore, there was no effect on VEGF-A expression in ARPE19 cells after the EV-mediated delivery of miRNA-466c enriched by hCD9.hAGO2. Similarly, shRNA-451, targeted to Vegf-a, and delivered by EVs, was not functional in C166 cells [259]. 

Another loading strategy has been developed based on the archaeal ribosomal protein L7Ae, conjugated with CD63 [108]. This system has been called EXOtic (exosomal transfer into cells). The packaging of mRNA is provided by L7Ae, which recognizes a C/D_box_ RNA structure, introduced into the 3′UTR of the particular mRNA. Additionally, EXOtic includes two other plasmids. The first provides an increased yield of EVs by encoding genes that potentially enhance the release of EVs. In particular, the combined expression of STEAP3, syndecan-4, and a fragment of L-aspartate oxidase in HEK293T cells, expressing CD63 fused to the NanoLuc (Nluc) bioluminescence protein, caused up to a 40-fold increase in the luminescence signal in the cell supernatant [108]. The increased production of EVs was confirmed by nanoparticle tracking analysis (NTA) without a change in the size distribution of EVs. The second plasmid in EXOtic was developed to facilitate the release of mRNA from EVs into the cytoplasm of recipient cells. For that, a constitutively active mutant S368A of a gap junction protein, connexin 43 (Cx43), potentially enriched in EVs, was used to enhance the transfer of mRNA from EVs to target cells through the formation of hexameric channels [108]. 

A glycoprotein, lactadherin, is considered another biological marker, presenting in EVs of some cell types [260]. Its C1C2 domains have also been extensively used for EV engineering. Recently, a capsid protein of phage MS2 was fused to C1C2 to load pre-miRNA-146a into EVs [261]. The MS2 capsid protein has interacted with binding sites, the RNA stem-loop structures, flanking the sequence of pre-miRNA-146a, providing its encapsulation and enrichment in EVs. The biological activity of miRNA-146a was preserved after the delivery by EVs in vitro and in vivo. 

The bacteriophage MS2 system was recently used along with another EV surface protein, LAMP-2B (lysosome-associated membrane protein 2 isoform B). The MS2 capsid protein was fused to the C-terminus of LAMP-2B, and the loading of target mRNA into EVs was provided via the interaction of the MS2 capsid protein with the binding site inserted into the 3′UTR of mRNA. Using this strategy, the authors constructed chimeric antigen receptor (CAR) T cells directly from PBMCs by their treatment with EVs bearing CAR-encoding mRNA and anti-CD3/CD28 scFv. The anti-CD3 scFv/anti-CD28 scFv were fused to the N-terminus of LAMP-2B that provided its display on the EV surface [262]. 

## 9. Direct (Exogenous) Strategies for tNA Loading into EVs

An alternative to the endogenous loading of tNAs is the direct loading of drug preparations into purified EVs. Some strategies are focused on the anchoring of tNAs in the membrane of EVs; others aim at the encapsulation of tNAs inside EVs. Overall, the exogenous loading can be quite “traumatic” for EVs [263]. As a rule, there is a tendency for EVs to form aggregates after various exposures. A potentially undesirable approach for tNA loading is sonication, since it can cause degradation of tNAs [264].

Here, we will discuss two of the most commonly used direct strategies for tNA loading into EVs. They are mainly adapted for EVs with a small size (up to ~200 nm).

### 9.1. Binding of tNAs with the EV Membrane

The association of tNAs with the surface of EVs seems less preferable than their intravesicular encapsulation. However, this approach may have prospects for nuclease-resistant tNAs. One of the methods providing for the efficient binding of tNAs with EVs is the bioconjugation of tNAs with lipophilic molecules [265]. The conjugation of antisense oligonucleotides (ASOs) [266], small interfering RNA (siRNA) [267], or miRNA [268] with cholesterol can ensure their spontaneous binding to the membrane of EVs. Thus, HepG2-derived EVs modified with an arginine-rich cell-penetrating peptide (R9) were used to deliver a cholesterol-modified ASO (G3139) targeted to Bcl-2 (B-cell lymphoma 2) into their own cells [266]. The R9 peptide was attached to EVs via covalent bond formation between carboxyl groups of the C-terminus of arginine and the amino groups of transmembrane proteins or PE. The treatment of HepG2 cells with EVs-R9-G3139 or EVs-G3139 suppressed Bcl-2-encoding mRNA by 72.8% and 44.3%, respectively [266]. Another research describes the loading of mesenchymal stromal cell-derived EVs by cholesterol-modified miRNA-210, applied to improve angiogenesis in the ischemic brain of mice [268]. Targeting of EVs was provided by conjugation with the peptide c(RGDyK) (cyclo (Arg-Gly-Asp-D-Tyr-Lys)), which binds with the integrin α_v_β_3_ in reactive cerebral vascular endothelial cells. This peptide was conjugated to EVs using bio-orthogonal copper-free click chemistry. The delivery of miRNA-210 by RGD-EVs induced angiogenesis in a lesion region in the mice’s brain. 

Cholesterol-containing siRNA (ch-siRNA) is the most studied class of hydrophobic tNAs (h-tNAs). Without a carrier, ch-siRNA forms complexes with lipoproteins in the bloodstream and is internalized by cells in this form via receptor-mediated endocytosis [269,270]. Unfortunately, ch-siRNA intensively accumulates in the liver [271,272] when administrated intravenously, which limits its clinical application. Carrier-mediated ch-siRNA delivery can partially solve this problem and change the ch-siRNA organotropism. In addition, other siRNA conjugates, for example, with docosanoic acid (DCA) [271,273], which are more suitable for extrahepatic delivery, can be used instead of cholesterol modification. Remarkably, DCA-siRNA and ch-siRNA demonstrated similar levels of binding to EVs [265].

The association of ch-siRNA with EVs may influence its distribution in a tissue. The study of the ch-siRNA biodistribution in the mouse brain after local administration showed that, without a carrier, ch-siRNA preferentially accumulated at the injection site [267]. In contrast, EVs allowed ch-siRNA to spread to both sides of the brain after unilateral administration. Moreover, it should be noted that the loading of ch-siRNA into EVs requires fine optimization because the insertion of too much ch-siRNA may adversely affect siRNA biological activity [274]. 

### 9.2. The Loading Using Transient Permeabilization of the EV’s Membrane

Increasing the permeability of the EV membrane can be achieved by different approaches, including techniques that are routinely used for bacterial cell transformation. The most common way to load tNAs into EVs is electroporation. Electroporation (also called electropermeabilization) is based on a damage of the structure of the EV membrane by an electrical impulse. It is believed that the damage is temporal, and the integrity of the EV’s membrane is restored after a short time.

The efficiency of tNA incorporation into the electroporated EVs depends on many parameters, including the size of the nucleic acid. “Small” tNAs are loaded into EVs more efficiently. The loading of “large” tNAs, such as pDNA, can cause serious difficulties [275]. Therefore, it was proposed to load EVs with minicircle (MC) expression cassettes, which are smaller than pDNA and have a prolonged action [276]. The delivery of anti-alpha-synuclein short hairpin RNA expression vector (shRNA-MC) by EVs to the central nervous system was analyzed in mice injected with alpha-synuclein preformed fibrils (Syn PFFs). Ninety days after Syn PFF injection, the administration of two doses of shRNA-MC-EVs resulted in a decrease in the alpha-synuclein aggregation in several brain regions. For targeting the brain, EVs were modified with the RVG peptide. 

The loading efficiency of “small” tNAs into EVs, according to some estimates, can reach ~5%–24% [277,278] at a voltage not exceeding 400 V. An enhancement of the EV loading can be achieved using higher voltages and/or a larger number of pulses [279,280,281]. However, it should be taken into account that the use of too “strong” parameters for electroporation may adversely affect the integrity of EVs [281]. Furthermore, it should be noted that there are some concerns regarding the overestimation of the tNA loading efficiency into the electroporated EVs due to the aggregation of both tNAs [282,283] and EVs [277,284]. 

Chemical destabilization of EVs is used less frequently than electroporation [285,286]. The change in the permeability of the EV membrane in this case is achieved by treatment with calcium chloride and temperature imbalance. Typically, EVs and tNAs are mixed, and CaCl_2_ is added at a final concentration of 0.1 M. The mixture is then placed on the ice for 30 min, followed by heat shock at 42 °C. It is thought that Ca^2+^ neutralizes the charge of both tNAs and EVs/cells and promotes the adsorption of tNAs on the membrane surface [287]. The low temperature restricts the fluidity of the EV membrane. In turn, the entry of tNAs into EVs/cells is mediated by the temperature imbalance aroused after heat shock exposure [287]. 

The calcium chloride–associated method is usually used to load “small” tNAs into EVs. Its potential for loading “large” tNAs is poorly understood. Apparently, the biological activity of tNAs can be well preserved by this approach. For example, the intratracheal administration of Myd88 siRNA loaded into serum-derived EVs by the calcium-associated method resulted in a significant decrease in *Myd88* mRNA in bronchoalveolar lavage fluid macrophages in LPS-pretreated mice [286]. Another research showed that miRNA-375-3p mimic loaded into tumor EVs reversed the epithelial–mesenchymal transition state of HT-29 and SW480 cells [288]. The viability of tumor-bearing mice treated with tumor-derived EVs loaded with miRNA-124-3p was significantly increased compared with control groups, including those treated with unloaded EVs [289]. All this indicates that EVs can transfer functional tNAs after Ca^2+^-mediated chemical destabilization.

Another way to chemically permeabilize the EV membrane is to use saponins, which make holes in the membrane by interacting with cholesterol, followed by its removal [290]. Apparently, our group was one of the few who used saponins to load tNA into EVs, although saponins are successfully used for loading other types of cargo [291,292]. We loaded short DNA oligonucleotides into EVs secreted by human mesenchymal stem cells of the functional layer of the endometrium [293]. Since we did not observe any benefit of using saponin over the other tested loading strategies, we abandoned its further use in favor of another approach. 

Freezing–thawing (Fr–Th) is our preferred approach for loading oligonucleotides into EVs and artificial vesicles [253,293]. This method is based on a temporary disruption of the integrity of the EV membrane. A study of the properties of giant unilamellar vesicles subjected to Fr–Th showed that a change in their membrane permeability may be associated with damage caused by the formation of ice crystals [294]. Subsequently, during thawing, the vesicle membrane can be restored. 

Fr–Th has three significant advantages: it does not require special laboratory equipment; makes it easy to standardize the experimental conditions; and facilitates the performance of studies in sterile conditions, without requiring additional preparation. We have found that the use of Fr–Th can be as efficient as sonication or permeabilization of the EV membrane with saponin [293]. According to another observation, the loading of RNPs into EVs by Fr–Th was ~2 times more efficient than the use of sonication [295]. It is noteworthy that Fr–Th does not increase the toxicity of EV preparations, which can occur after the application of saponins, and also eliminates the risk of uncontrolled heating of samples, as in the case of sonication. 

The main limitation of Fr–Th is that temperature jumps can cause the aggregation or destruction of EVs. Interestingly, EV stability seems to be highly dependent on the cellular source of the vesicles and Fr–Th conditions. For example, EVs secreted by human erythrocytes showed high stability after the first, second, and even third freezing cycles [277]. On the contrary, EVs obtained from the blood plasma of healthy donors were susceptible even to a single exposure. Their number decreased already after the first freezing, in parallel with an increase in the size of EVs [296]. A slight (~10%) decrease in the amount of EVs derived from brain endothelial cells (bEnd.3) was observed after single or double freezing at −20 or −80 °C [297]. More pronounced changes (~15–20%) were observed upon the freezing of EVs in liquid nitrogen. After the third cycle of Fr–Th, the trend towards a decrease in the amount of EVs persisted under any freezing conditions. Again, this change was the most pronounced in the case of the use of liquid nitrogen, and the amount of EVs decreased by ~50% [297]. It should be noted that optimization of the composition of the storage buffer can have a beneficial effect on EV stability [298,299].

## 10. Clinical Trials and Some Recent Patents Related to the EV Application for tNA Delivery

EVs have become of considerable interest in medicine and are being used in a wide range of clinical trials [17,48,263,300,301,302,303]. While the focus is on the application of EVs for diagnostic and regenerative purposes, three clinical trials are aimed at the delivery of tNAs by EVs (Table 1; https://www.clinicaltrials.gov; 3 April 2023).

The NCT03608631 trial is planned to include up to 28 patients, 18 years and older, with histologically confirmed metastatic pancreatic ductal adenocarcinoma harboring the KrasG12D mutation. Participants will receive three doses of mesenchymal stromal-cell-derived EVs, loaded with KrasG12D siRNA. The treatment is planned to be repeated every 14 days for up to three courses with a possible continuation of three additional courses. This trial is scheduled to be completed in 2023.

According to www.clinicaltrials.gov (3 April 2023), the status of NCT03384433 is still unknown. However, it appears that there was some testing performed between January 2019 and September 2020. The safety of EVs derived from mesenchymal stem cells of the human placenta was evaluated after intraparenchymal implantation in five men with malignant middle cerebral artery infarct and a mean age of 62 years [304]. At the same time, the enrichment of EVs with miRNA-124 was not carried out during this trial, and the tests were performed with unmodified EVs. However, it should be noted that MSC EV-based therapy was well tolerated. One of the patients died 7 days after surgery due to the extension of ischemia, whereas the others had no complications after therapy and even led to an improvement in some functional parameters.

The NCT05043181 trial was scheduled to start in December 2021. It included 30 people aged 18–45 years with homozygous familial hypercholesterolemia (HoFH) diagnosed by genetic testing. The low-density lipoprotein receptor (ldlr)-encoding mRNA-enriched EVs will be injected into patients through abdominal puncture under ultrasound guidance. The estimated completion date for this clinical trial is December 2026.

To overcome the challenges associated with tNA loading into EVs, attempts are constantly being made to develop new loading technologies. Among them, it has recently been proposed to use the Arc (activity-regulated cytoskeleton-associated) protein for designing an mRNA delivery system (Patent #WO2022235723A1). It has been shown that the Arc protein self-assembles into spheroid particles that can carry mRNA inside them. Moreover, these particles are enveloped by EVs and transferred to recipient cells. 

Another group of inventors proposed a simple loading approach based on the conjugation of tNAs with cell-penetrating polypeptides (Patent #US20220265843A1). The loading process looks like a coincubation of EVs with peptide-tNA conjugates. Thus, miRNA-21-5p was recently covalently conjugated with a cell-penetrating peptide, YARA (YARAAARQARA), derived from the protein transduction domain of the human immunodeficiency virus-1 transactivator of transcription (HIV-1 Tat) [305]. Conjugation was carried out by forming a disulfide bond between YARA, bearing at the C-terminus an additional residue of glycine and cysteine, and miRNA-21-5p containing a 5′-thiol moiety. The presence of YARA enhanced the loading of miR-21-5p into EVs by 18.6-fold.

An intriguing question arises in the context of a recent invention about the type of transcripts sorted into EVs. This research aims to develop a tNA loading system using a pre-miRNA-451 structural mimic (Patent #US10851372B2). Interestingly, the replacement of the miRNA-451 sequence in its precursor (pre-miRNA) with the siRNA of interest can provide siRNA enrichment in EVs [283]. Apparently, a hairpin structure of pre-miRNA has been proposed as more important than the tNA sequence for packaging into EVs [283], indicating that at least miRNA can sort into EVs as a precursor. 

Among the approaches that have been implemented for a long time but continue to be actively developed, one can single out the creation of hybrids between EVs and lipid particles. Thus, milk vesicles (including EVs) were recently applied to form hybrids with cargo-bearing lipid particles for oral delivery (Patent #WO2021142336A1). This method can overcome the disadvantages of lipid particles and EVs when used alone as tNA delivery systems [306]. The advantage of this approach is that lipid particle loading strategies tend to be standard, unlike EVs [306]. Simultaneously, EVs can provide tissue specificity for the hybrid system. Moreover, milk-derived EVs can protect the content of the hybrid from degradation in the digestive tract.

A key limitation of the hybrid system is the difficulty in interpreting the data obtained after its application due to the problem with hybrid separation [307,308]. Nevertheless, some tools make it possible to evaluate the fact of hybrid formation after coincubation of EVs and lipid particles. One convenient solution is to use mesenchymal stem cells as a test model that are resistant to liposome (Lipofectamine 2000)-mediated transfection [309]. During the delivery of large tNAs, small-size EVs are also not efficient in the transfection if used alone. Thus, if large tNAs are delivered to mesenchymal stem cells, only hybrids formed between EVs and liposomes can provide this [309].

## 11. Conclusions

The results of numerous studies suggest that EVs can be considered to be promising tNA carriers, combining biocompatibility and the possibility of targeting, but their practical applications are currently limited due to the lack of cost-effective and large-scale technologies for their production, as well as the lack of efficient methods for tNA loading into EVs. One possibility to bring EVs to the bedside is the engineering of artificial mimetics of EVs that can be produced under standardized conditions in large quantities and loaded with tNAs. The development of methods for stimulating EV secretion is also of practical interest; however, the composition of these vesicles can be changed as a result of exposure, so the properties of these EVs must be studied separately. In addition, it is possible that the search for a suitable biological source of EVs will make their mass clinical application more feasible using already-existing isolation approaches. The endogenous loading of EVs can become a method of choice and of practical importance when the mechanisms underlying the EV formation will be elucidated, yielding the knowledge needed for manipulating them. The development of strategies for selective endogenous loading of EVs can both increase the efficiency of tNA incorporation and reduce therapeutic doses of EVs.

## Figures and Tables

**Table 1:** Clinical trials of EVs aimed at tNA delivery.

NCT Number	Trial Phase	Conditions	Cargo	Status	Location
NCT03608631	I	Pancreatic cancer with KrasG12D mutation	KrasG12D siRNA	Recruiting	United States
NCT03384433	I, II	Cerebrovascular disorders	miRNA-124	Unknown	Iran
NCT05043181	I	Homozygous familial hypercholesterolemia (HoFH)	Low-density lipoprotein receptor (ldlr)-encoding mRNA	Not yet recruiting	China

## Data Availability

Not applicable.

## Abbreviations

ALIX—ALG-2-interacting protein X; ARRDC1—arrestin domain-containing protein 1; ASO—antisense oligonucleotide; ch-siRNA—cholesterol-containing siRNA; ESCRT—endosomal sorting complex required for transport; EVs—extracellular vesicles; Fr–Th—freezing–thawing; h-tNAs—hydrophobic tNAs; ILVs—intraluminal vesicles; miRNA—microRNA; MVB—multivesicular body; NA—nucleic acid; pDNA—plasmid DNA; PE—phosphatidylethanolamine; PS—phosphatidylserine; RBP—RNA-binding protein; RNP—ribonucleoprotein; sgRNA—single-guide RNA; shRNA—short hairpin RNA; siRNA—small interfering RNA; snoRNA—small nucleolar RNA; tNAs—therapeutic nucleic acids.
